# Artificial Neural Network to Forecast Enhanced Oil Recovery Using Hydrolyzed Polyacrylamide in Sandstone and Carbonate Reservoirs

**DOI:** 10.3390/polym13162606

**Published:** 2021-08-05

**Authors:** Hossein Saberi, Ehsan Esmaeilnezhad, Hyoung Jin Choi

**Affiliations:** 1 Department of Petroleum Engineering, Hakim Sabzevari University, Sabzevar 9617976487, Iran; Hossein.saberi1998@gmail.com; 2 Department of Polymer Science and Engineering, Inha University, Nam-gu, Incheon 22212, Korea; 3 Program of Environmental and Polymer Engineering, Inha University, Nam-gu, Incheon 22212, Korea

**Keywords:** polymer, enhanced oil recovery, artificial neural network, fuzzy logic

## Abstract

Polymer flooding is an important enhanced oil recovery (EOR) method with high performance which is acceptable and applicable on a field scale but should first be evaluated through lab-scale experiments or simulation tools. Artificial intelligence techniques are strong simulation tools which can be used to evaluate the performance of polymer flooding operation. In this study, the main parameters of polymer flooding were selected as input parameters of models and collected from the literature, including: polymer concentration, salt concentration, rock type, initial oil saturation, porosity, permeability, pore volume flooding, temperature, API gravity, molecular weight of the polymer, and salinity. After that, multilayer perceptron (MLP), radial basis function, and fuzzy neural networks such as the adaptive neuro-fuzzy inference system were adopted to estimate the output EOR performance. The MLP neural network had a very high ability for prediction, with statistical parameters of R^2^ = 0.9990 and RMSE = 0.0002. Therefore, the proposed model can significantly help engineers to select the proper EOR methods and API gravity, salinity, permeability, porosity, and salt concentration have the greatest impact on the polymer flooding performance.

## 1. Introduction

After primary production, approximately two-thirds of the initial oil in place is expected to remain in the reservoirs. Enhanced oil recovery (EOR) methods which have become a main subject in petroleum engineering to meet the demand for energy will extract enough oil to fulfill a significant portion of the global oil demand [1]. As an EOR method, chemical flooding has been a popular strategy for improving oil recovery in mature oil fields that is now carried out using a variety of chemical agents and it has been shown to be successful [2]. Two types of features, microscopic and macroscopic sweep efficiencies, are considered in an EOR process. For the first case, chemical agents like surfactants are used and, for the second one, polymers are utilized to improve the mobility ratio by increasing the shear viscosity of water. Polymer flooding is an effective way to boost the water flooding effect and field experiments and applications have been conducted in a series of oil fields, with positive results in terms of increasing oil production [3,4], where water-soluble polymers were used to improve the rheological properties of water [5]. Therefore, every factor that strengthens or weakens rheological properties of the polymer solution is an influential factor [6]. Besides the polymer type and its concentration, there are many influencing factors which should be considered regarding the water, oil, and rock type of a reservoir [7]. Therefore, the various screening criteria of polymer flooding make the evaluation of its performance before field-scale operations difficult. One way to overcome this issue is the simulation of the process through core flood experiments that are still expensive and time consuming. One more way that is more economical and facile is using simulation tools such as an artificial neural network (ANN), fuzzy inference system (FIS), evolutionary computation (EC), and their hybrids, which have all been used effectively to construct a predictive model [8]. These methods are appealing because they can deal with various uncertainties. Soft computing approaches are increasingly employed as a substitute for traditional statistical methods [9]. To the best of our knowledge, the modeling and prediction of the polymer flooding experiment have not been widely investigated, particularly using ANNs such as multilayer perceptron (MLP), radial basis function (RBF), and fuzzy neural networks such as an adaptive neuro-fuzzy inference system (ANFIS). The current study investigated the performance of polymer flooding by using the abovementioned modeling tools. The first step is to find out the important factors and, as mentioned above, the polymer type and its concentration are known as influential factors. The most widely used polymer in petroleum engineering for EOR operations is hydrolyzed polyacrylamide (HPAM). Therefore, its data are shown in this article [10,11]. In addition to the species and concentration of polymer and its molecular weight, both the type and concentration of salt also have a great effect on the rheological properties [12] because the addition of divalent ions causes a large decrease in the rheological properties of the polymer solution [13]. Hence, in addition to the salt concentration, three categories are considered regarding salt type. The first category is fresh water, the second category is a low saline, which is assigned to monovalent salts, and the third category is a high saline, which is assigned to salts that contain both monovalent and divalent ions [6]. Mobility ratio, which is roughly defined as the ratio of flooded fluid (oil) to flooding fluid (polymer solution) [14], is an important influencing parameter that should be considered during performance evaluations of EOR processes. As mentioned previously, the viscosity of polymer solutions is dependent on several parameters that are considered as inputs for the models and, therefore, it cannot be considered directly because putting dependent parameters as inputs in the model will impose huge complexity on the model versus fewer gains. Hence, some of the abovementioned independent parameters, of which the polymer solution viscosity is a function, were selected as inputs to indirectly see the effect of polymer solution viscosity. Additionally, as for the oil viscosity, because it is indirectly dependent on American Petroleum Institute (API) oil gravity [15,16,17], just the API gravity was considered as an input parameter to avoid complexity.

Not only rock type [18], porosity [19], permeability [20], temperature [21], API gravity [22], and initial oil saturation [23], among the reservoir properties, but also the volume of flooded fluid (pore volume (PV)) among the operational parameters, were considered as input parameters for the ANN and, finally, EOR was predicted using the abovementioned networks [24].

Briefly, the aim of this paper is to introduce a proper model with high accuracy to predict the performance of polymer flooding as an EOR method before doing any lab- and field-scale activities.

## 2. Methodology

### 2.1. Data Collection

Six prior investigations on both carbonate and sandstone core reservoir samples provided the raw data needed for modeling [25,26,27,28,29,30]. There were 847 data records in the gathered data sets, which were separated into three groups: training (70%), validating (15%), and testing data (15%). Eleven relevant elements were present in the actual or experimental input data, including (1) polymer concentration, (2) salt concentration, (3) rock type, (4) initial oil saturation, (5) porosity, (6) permeability, (7) pore volume flooding, (8) temperature, (9) API gravity, (10) molecular weight of the polymer, and (11) salinity. The only output of the utilized models was the oil recovery factor via polymer flooding compared to the final one after pure water flooding per unit percentage (%), which was presented as a percentage and dubbed “EOR after polymer flooding”. Table 1 displays the ranges of various input parameters.

### 2.2. ANN

ANNs, often known as neural networks, are current systems and computational approaches for machine learning, knowledge presentation, and, lastly, using that information to maximize the output responses of complex systems. The primary principle behind these networks is based on how the biological brain system processes data and information to learn and produce knowledge. The creation of new methods for information processing systems is a major component of this concept [31].

This system consists of a huge number of highly linked processing components, i.e., neurons that collaborate to solve problems and send information via synapses (electromagnetic communications). If one cell in these networks is harmed, other cells can be compensated by contributing to its regeneration. Thereby, these networks can learn. By injecting tactile nerve cells, for example, the cells learn not to travel to the heated body, and the system learns to fix its error with this algorithm. These systems learn adaptively, meaning that when new inputs are presented, the weight of the synapses changes in such a manner that the system delivers the proper response [32].

Input, output, and processing are the three levels of an ANN unless the user inhibits communication between neurons, and each layer comprises a set of nerve cells that are ordinarily interconnected with all nerve cells in other layers. However, the nerve cells in each layer have no link with other nerve cells in the same layer. A nerve cell is the smallest unit of information processing that allows neural networks to operate. A neural network is a collection of neurons that build a specific architecture based on connections between neurons in distinct layers while being positioned in distinct layers. As neurons are a type of nonlinear mathematical function, a neural network made up of them can be a fully complicated nonlinear system. Each neuron in a neural network works independently, and the network’s overall activity is the product of the actions of numerous neurons. In other words, neurons in a cooperative process correct each other [33,34,35].

### 2.3. MLP Artificial Neural Network

The MLP network consists of several types of layers, including the input, one or more hidden layers, and the output that every type of layer possesses some of the processing neurons, and every neuron is entirely linked to succeeding layers via a weighted interconnection [8]. Therefore, the first one has an equal number of input parameters and neurons, and the model’s output is related to a neuron in the third one. Additionally, the correlation of the model’s output and input is specified in the second type of layer. Their numbers of hidden layers and neurons will crucially affect the efficiency of the MLP network [36]. The node’s value in the second and the last type of layer is determined based on its weight in the former layer [37]. After that, the offset value is aggregated to the gained results, and the computed value is transited to the trigger level via the transfer function to generate the final output. Various activation functions, such as a binary step, identity Gaussian, and linear functions, could be adopted for the second and third types of layers. The following equation shows the results of the model:

(1) $$y_{k}=F_{k}\left(\overset{m}{\underset{i=1}{\sum }}w_{kj}x_{j}+b_{k}\right)$$

where $y_{k}$ is the output, $w_{kj}$ is the link weight, $x_{j}$ is the input, $b_{k}$ is the bias vector, and $F_{k}$ is the activation function. The MLP training process is executed using a backpropagation algorithm such as scaled conjugate gradient, gradient descent, Levenberg–Marquardt, and resilient backpropagation [38].

**Figure 1:**
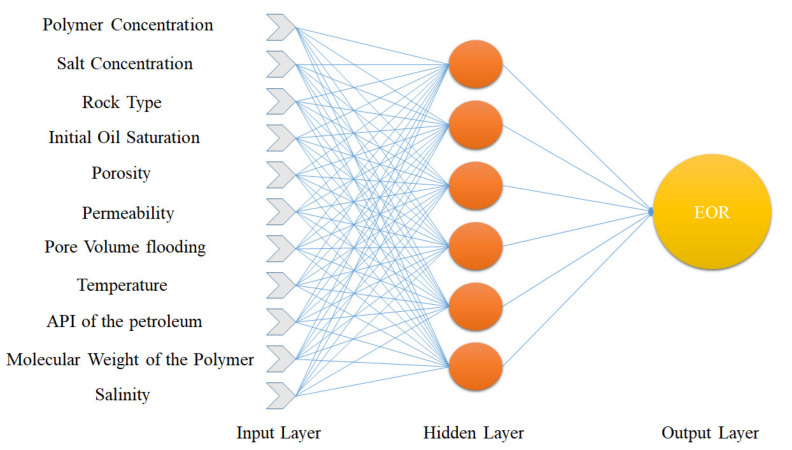
MLP network structure for EOR prediction.

In this paper, among several activation functions used in MLP artificial neural networks including tangent sigmoid (*tansig*) and log-sigmoid, and linear transfer function (*purelin*), *tansig* is used for the link between the input and the hidden layers and *purelin* is adopted for the link between the hidden and the output layers [8]. The structure of the MLP network used in this paper to predict the target data is shown in Figure 1.

### 2.4. Radial Basis Function (RBF) Artificial Neural Network

The feasibility of this kind of neural network to process arbitrary sparse data, that is easy to generalize to multidimensional space, and to provide spectral precision makes it a particularly suitable alternative [38]. In addition, the RBF neural network is superior to the MLP model because it has excellent accuracy in nonlinear data modeling and can be trained in a single direct program instead of an iterative solution in MLP [39]. While the frame of RBF is comparable to the MLP [40], the RBF possesses only one hidden layer, which consists of multiple nodes called RBF units. The RBF neural network architecture is a two-layer feed-forward neural network, in which the input is transmitted from the neurons in the hidden layer to the output layer. Each RBF network possesses two important factors, which describe the center position of the function and its deviation. Finding the center of the unit and determining the optimal value of the weight connecting the RBF unit and the output unit are the two main steps in the training process of the RBF neural network [41]. Different methods, such as random center selection [42], clustering [43], and density estimation [44], could be adopted to discover the center in the RBF network. The output of the system can be expressed as Equation (2):

(2) $$f\left(x_{i}\right)=w^{T}\varphi \left(x_{i}\right)$$

where $w^{T}$ is the transposed output of the shell vector, and $\varphi \left(x_{i}\right)$ is a kernel function. For this, different optimization algorithms can be applied. Here, trial and error are adopted to find out the optimal value for this parameter [8]. By changing this parameter, RBF neural networks with different structures are developed and the product of each RBF neural network is observed according to the MSE value of the test data subset [38].

### 2.5. ANFIS

This fuzzy logic (FL)-based average value was initially provided in 1998 [45]. The ANFIS model can use qualitative methods to solve nonlinear problems and model physics, instead of operating quantitative methods by turning input data into a particular term called even fuzzy set or linguistic. The frame of the neuro-fuzzy system has five layers which are illustrated here [46,47].

First, the fuzzy input is made based on transforming input data by defining a membership function (MF) [48]. The computed membership degree of every input factor is reproduced, resulting in the firing strength, as shown below:

(3) $$w_{i}=\overset{m}{\underset{i=1}{\prod }}\mu_{ij}\left(x_{j}\right)$$

where $w_{i}$ is the calculated firing strength, $\mu_{ij}$ is the degree of membership of the jth MF for the ith input, and *m* employs the input counter. For each rule, the firing strength is obtained using multiplication, and the highest one obtained matches with the input [32]. The next layer operates based on the following equation:

(4) $$\bar{w_{l}}=\frac{w_{i}}{\sum_{i}w_{i}}$$

where $\bar{w_{l}}$ is the normalized firing strength. At the end, the final result is obtained using the following equation:

(5) $$\bar{w_{l}}\times f_{i}=\bar{w_{l}}\times \left(\overset{m}{\underset{i=1}{\sum }}n_{ij}x_{j}+r_{ij}\right)$$

In the above formula, $f_{i}$ can be a constant or polynomial function. The values of $n_{ij}$ and $r_{ij}$ are the adjustment factors of TSK-FIS and their values should be optimized by the specified algorithm to have a more accurate prediction [8]. The ultimate layer sums the outputs in the prior layer to generate the following general ANFIS output:

(6) $$overall\;output=\underset{i}{\sum }{\bar{w}}_{i}f_{i}=\frac{\sum_{i}w_{i}f_{i}}{\sum_{i}w_{i}}\;$$

In this work, three types of ANFIS are studied, and the characteristic that distinguishes them is the distribution of membership functions. The first kind of system is identified by the division of the grid, and the membership function is uniformly distributed in space, while the second kind of system uses a subtractive grouping mechanism; the last one is based on fuzzy clustering of c-means. See a previous article for more theories [8].

To optimize the network parameters, the grasshopper optimization algorithm (GOA) [49], genetic algorithm (GA) [50], and swarm optimization algorithm (PSO) [51] can be applied on artificial neural networks. In this study, we used a trial and error method that examined 500 replications for each parameter to ensure that the optimal value of the data also changed randomly with each iteration.

## 3. Model Evaluation

Several statistical standards were adopted to evaluate the accuracy of the applied model, including coefficient of determination (*R*^2^), mean squared error (MSE), root mean squared error (RMSE), mean error (μ), error standard deviation (σ), and average absolute relative deviation (AARD):

(7) $$R^{2}=1-\frac{\sum_{i=1}^{N}{\left(EOR_{i}^{actual}-EOR_{i}^{predicted}\right)}^{2}}{\sum_{i=1}^{N}{\left(EOR_{ave}^{actual}-EOR_{i}^{predicted}\right)}^{2}}$$

(8) $$\mathrm{MSE}=\frac{1}{N}\overset{N}{\underset{i=1}{\sum }}{\left(EOR_{i}^{actual}-EOR_{i}^{predicted}\right)}^{2}$$

(9) $$\mathrm{RMSE}=\sqrt{\mathrm{MSE}}$$

(10) $$Error_{i}=EOR_{i}^{actual}-EOR_{i}^{predicted}$$

(11) $$\mu =\frac{1}{N}\overset{N}{\underset{i=1}{\sum }}|Error_{i}|$$

(12) $$\sigma =\sqrt{\frac{1}{N-1}}\overset{N}{\underset{i=1}{\sum }}\left|Error_{i}-\lambda \right|$$

where λ is the mean of the error:

(13) $$\lambda =\frac{1}{N}\overset{N}{\underset{i=1}{\sum }}Error_{i}\;$$

(14) $$\mathrm{AARD}=\frac{100}{N}\overset{N}{\underset{i=1}{\sum }}|\frac{EOR_{i}^{actual}-EOR_{i}^{predicted}}{EOR_{i}^{actual}}|$$

N is the amount of data, and $EOR_{i}^{actual}$ and $EOR_{i}^{predicted}$ represent the original target data and the predicted output of the model, respectively.

## 4. Results and Discussion

Among the 847 datapoints collected for each of the parameters ((1) polymer concentration, (2) salt concentration, (3) rock type, (4) initial oil saturation, (5) porosity, (6) permeability, (7) pore volume flooding, (8) temperature, (9) API gravity, (10) molecular weight of the polymer, (11) salinity, and (12) EOR) from different articles, an attempt was made to model using MLP, RBF, and ANFIS neural networks. In the following, the networks are created and their effective parameters and optimization are examined.

### 4.1. Optimum MLP Structure

The results of MLP neural network sensitivity analysis are shown in Table 2. This three-layer network, that includes input, hidden, and output layers, was evaluated with different training algorithms, due to their higher speed (which is less costly to the system) and higher efficiency (statistical parameters indicate this). The Levenberg–Marquardt backpropagation algorithm, as the superior algorithm, was used in the rest of the comparisons in this study (Table 3). In this study, we assigned 70% of the collected data to the training data and, to the validation and test data, 15%, which is clearly shown in Table 4, using this data distribution to obtain the power of the most optimal MLP networks. Additionally, based on Table 2, the best number of neurons was determined, and based on this, six neurons was considered to be the best. As the number of neurons in the MLP network increases, the quality and efficiency of the network increases, but more than six neurons in the data of this study do not increase the cost of efficiency, so six neurons are regarded to be the best value. The structure of the superior MLP network can be found in Figure 1.

**Figure 2:**
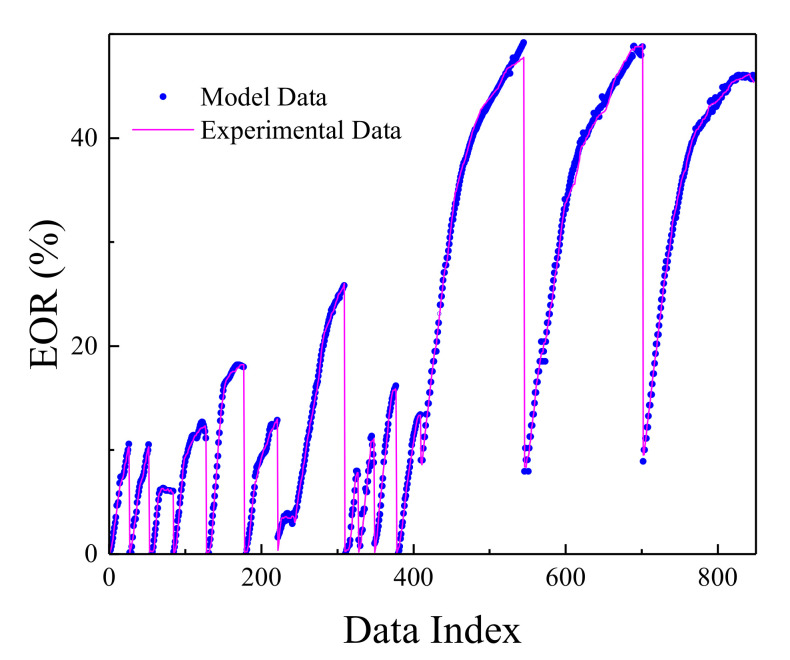
Comparison between actual EOR and MLP values.

**Figure 3:**
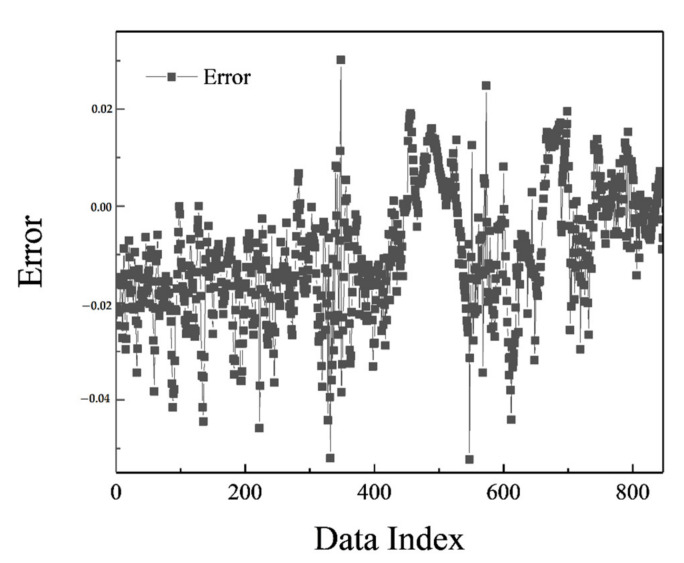
MLP error chart.

The MLP network was trained in the most optimal mode presented, and its result can be seen in Figure 2. Figure 3 shows the network error after data normalization, which is a reasonable and very low network error that can have many industrial applications in this field.

**Figure 4:**
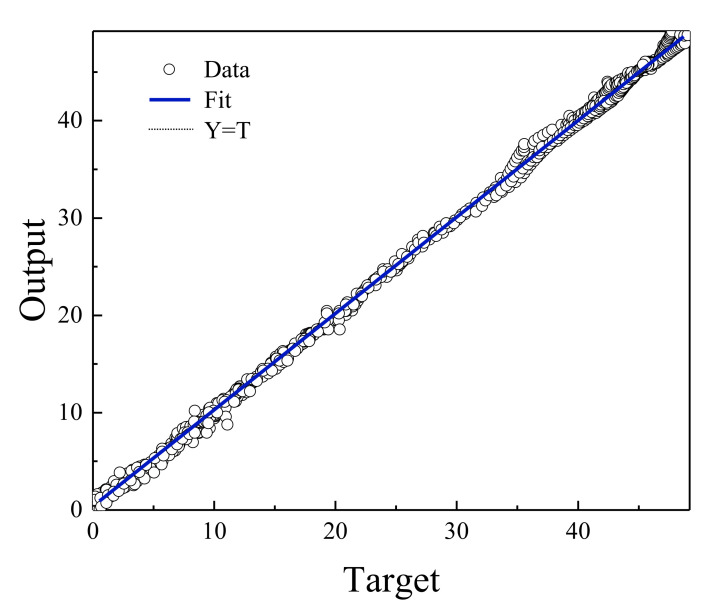
Relationship between MLP network output and EOR target data.

**Figure 5:**
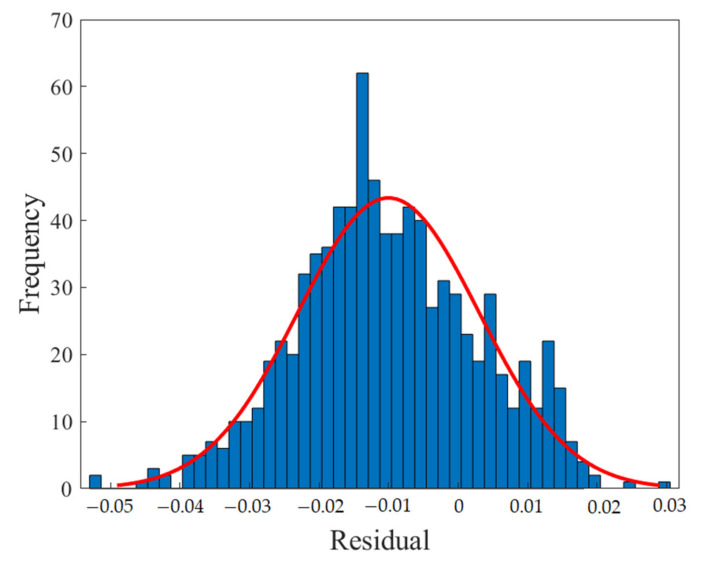
Error histogram of the MLP neural network.

Figure 4 shows the regression diagram of all the output data of the model and the target data obtained from the articles. This diagram shows the amount of difference between the model and target data that overlap. Figure 5 represents an error histogram graph that has been obtained from the normal data loss, which can also be seen below the network error value. Meanwhile, it can be seen that there are very few outliers that can easily be seen at this high level of precision.

### 4.2. Optimum RBF Structure

Based on Table 5, to determine the optimal parameter of the RBF neural network, we first determined the optimal maximum number of neurons in the hidden layer. After running the program about 700 times for each neuron from 1 to 100, 44 neurons were selected as the best number of neurons. Then, the spread coefficient was determined and this operation was carefully examined with about three points from 1 to 100, which gave the best network results with a spread coefficient of 1.1, which can be clearly seen in Table 5.

It should be noted that in this particular data, when increasing the spread coefficient, the accuracy of the network decreases, and also, as mentioned earlier, when increasing the number of neurons, more accuracy can be expected from the neural network, but this happens until the cost that is applied to the network is at the same level as the accuracy of the network (increasing neuron number increases the cost).

**Figure 6:**
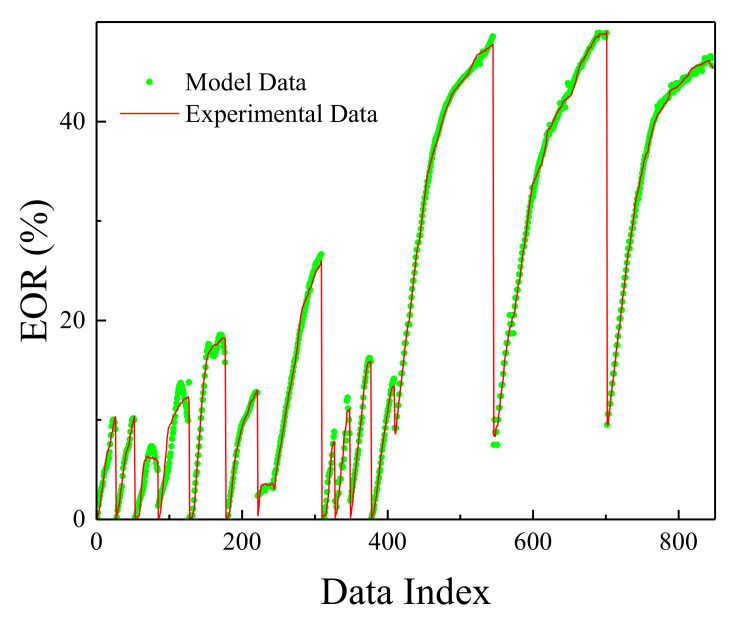
Comparison between actual EOR and RBF values.

**Figure 7:**
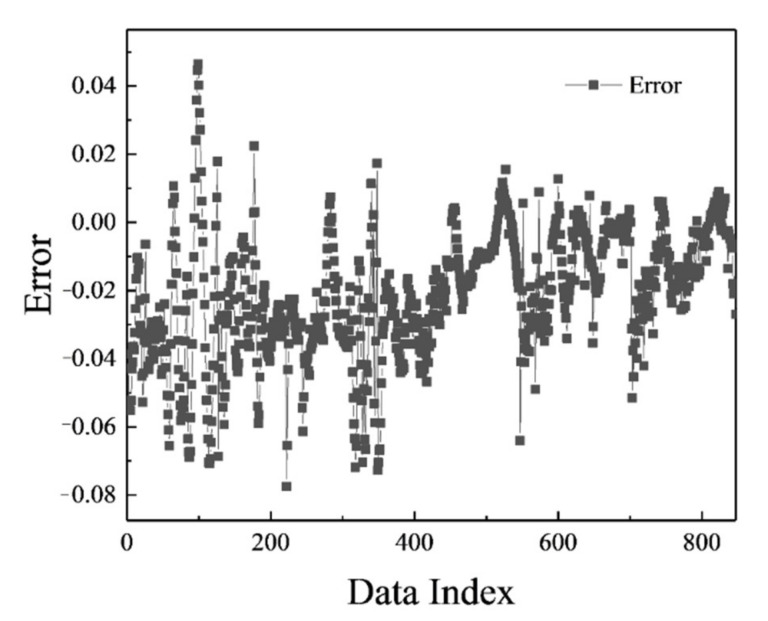
RBF error chart.

**Figure 8:**
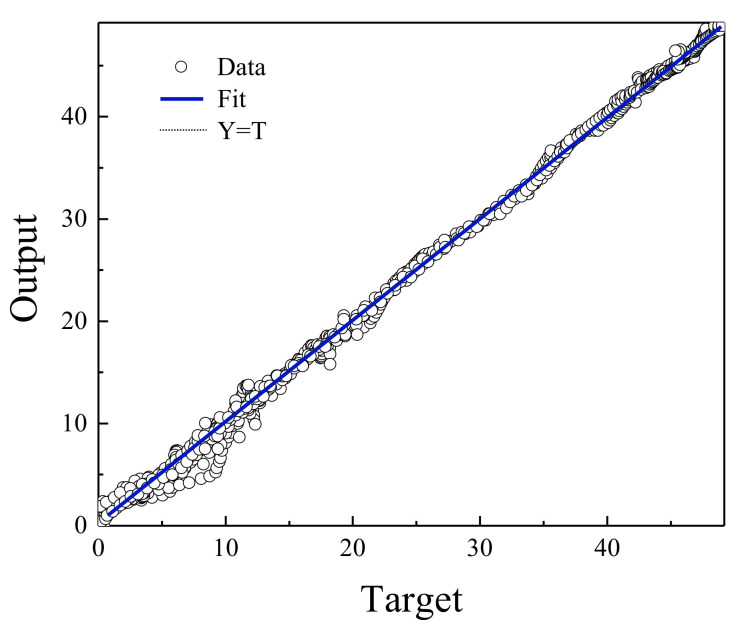
Relationship between RBF network output and EOR target data.

**Figure 9:**
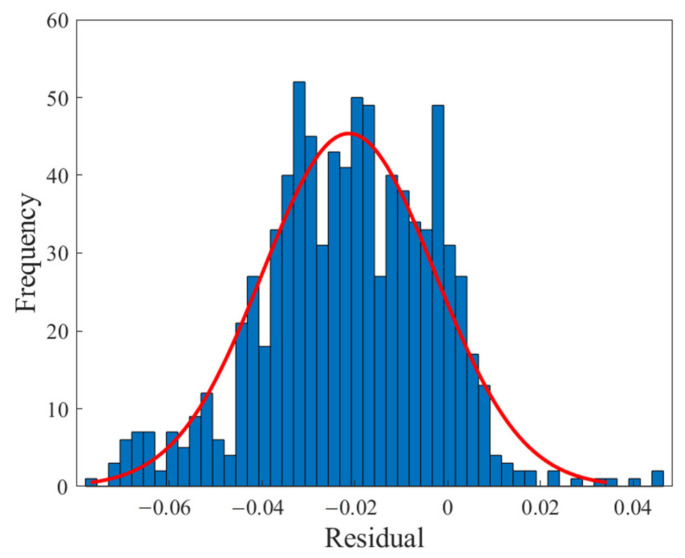
Error histogram of the RBF neural network.

Figure 6, Figure 7, Figure 8 and Figure 9 are related to the superior RBF neural network in polymer data, expressing the high accuracy of this neural network. Figure 6 and Figure 7, which show the normalized error of the data, indicate that this network has a very low error in data estimation and has a good ability to predict the data. In addition, based on Figure 8 and Figure 9, this capability can be seen and it can also be easily seen that this network has very few outliers and residual values have normal scattering, which show the strength of this network.

### 4.3. Optimum ANFIS Structure

Experiments were performed and different ANFIS networks from three common types of ANFIS, grid partitioning-based ANFIS, subtractive clustering based ANFIS, and fuzzy c-means (FCM) clustering, were run [8], and Table 6 shows their sensitivity analysis. Information about fuzzy neural networks based on subtractive clustering is reported.

To determine the optimal parameters of the fuzzy neural network which are presented in this section, a trial and error method and its repetition for each parameter at a rate of 100 times and recording network data and determining the best values for the neural network was carried out. Based on the sensitivity analysis that is shown in Table 7, it can be stated that by increasing the step size decrease rate for polymer data to 23, the desired result is obtained, and as the size of the network increases, an error also occurs. The same is true for changing the step size increase rate parameter up to 20. Increasing the value of the initial step size parameter above four and decreasing it to less than four networks do not provide the desired results. The value of the radius parameter is a vector that determines the range of influence of the center of the clusters in each of the data dimensions. With a lot of trial and error, a value of 0.333 was determined for this parameter, which provides the most desirable network for these polymer data.

**Figure 10:**
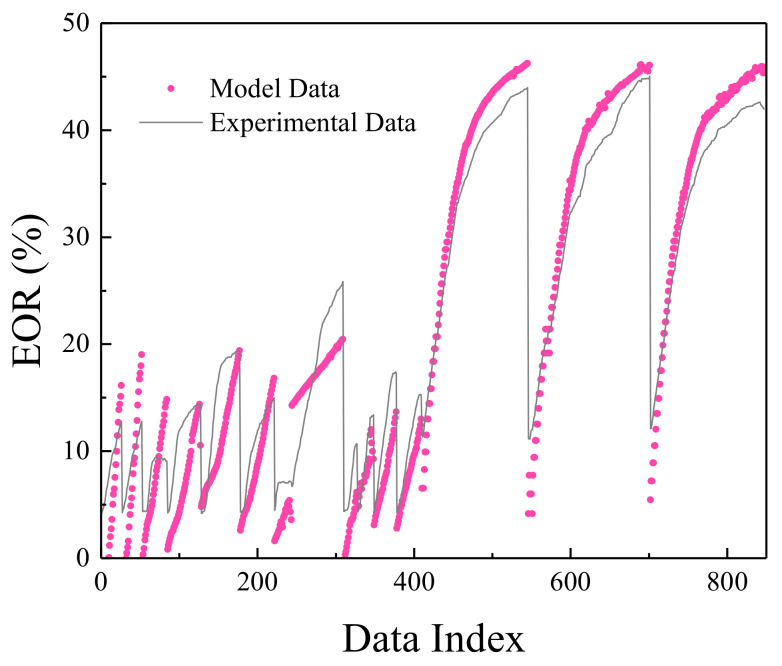
Comparison between actual EOR and ANFIS values.

**Figure 11:**
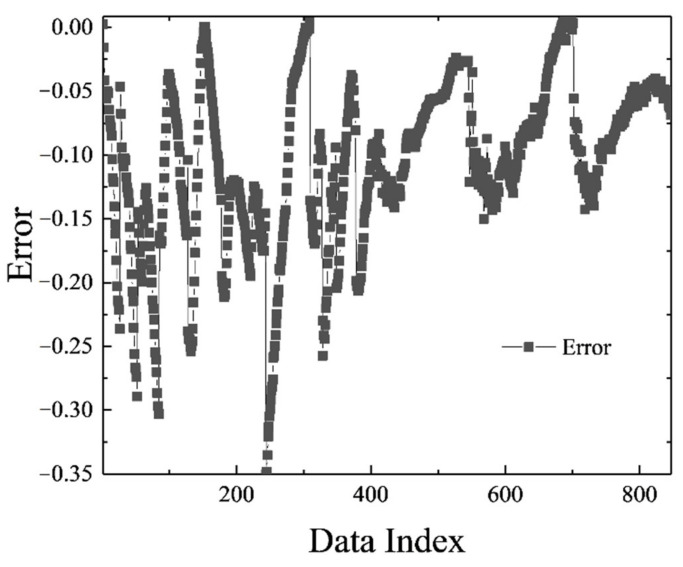
ANFIS error chart.

**Figure 12:**
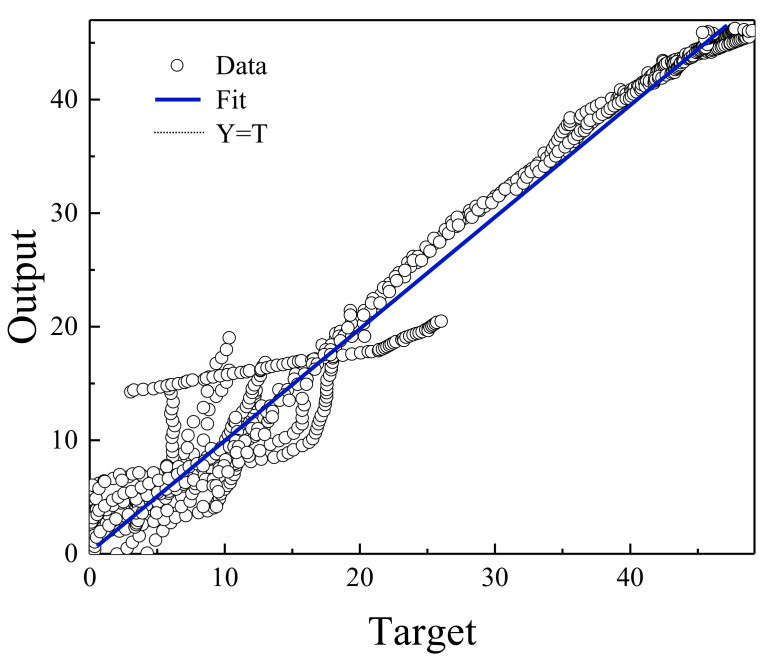
Relationship between ANFIS network output and EOR target data.

**Figure 13:**
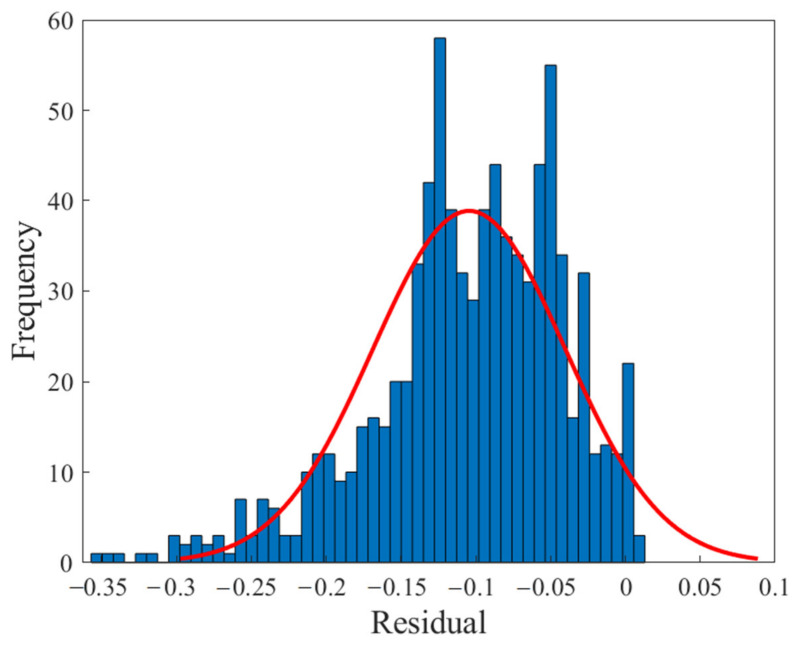
Error histogram of the ANFIS neural network.

Figure 10, Figure 11, Figure 12 and Figure 13 show the accuracy of this network, and according to Figure 10 and Figure 11, this network has more errors than the MLP and RBF networks presented in the previous sections. Based on Figure 12 and Figure 13 showing the linear regression and a histogram of the error of the data after their normalization, respectively, it was found that there are very few outlier when estimating data using the fuzzy neural network and relatively good accuracy but due to the higher accuracy seen in previous cases, it is less accurate than MLP and RBF neural networks.

### 4.4. Performances of Optimized MLP, RBF, and ANFIS Models

Based on the comparisons made in the previous sections, it can be presented that the MLP neural network has the best performance for teaching this type of data. Figure 2, Figure 3, Figure 4 and Figure 5 show the performance of the trained network using polymer data, which was discussed in detail in the previous sections. The MLP neural network with the Levenberg–Marquardt backpropagation algorithm, along with its sensitivity analysis presented in Table 2, has a very strong prediction ability with the desired data inside and outside the predicted range. The general shape of the network with six neurons in the hidden layer is shown in Figure 1. The complete information of the best-trained network (which is of the MLP type) is shown in Table 8.

## 5. Overfitting Evaluation

Overfitting is a phenomenon in which the accuracy of network training data is very high and powerful, but this is not observed in network test data. The reasons for this can be a small dataset [52] and a very complex model [53]. The figures and diagrams embedded in the previous sections clearly show that the trained neural networks do not involve overfitting at all, but a method similar to the Tabaraki and Khodabakhshi method presented in 2020 [53] can be used to prove that the models that are presented in this section do not include overfitting

At the beginning of the work for the target network (here, for the top MLP, RBF, and ANFIS networks), the value of the total number of adjustable parameters (TNAP) was calculated, and for this purpose the following equation is used:

(15) $$TNAP_{ANNs}=n_{hid}\times \left(n_{inp}+3\right)$$

(16) $$TNAP_{ANFIS}=\left(n_{inp}\times n_{mf}\times n_{pmf}\right)+\left(n_{out}\times n_{r}\right)$$

where $n_{hid}$, $n_{inp}$, $n_{mf}$, $n_{pmf}$, $n_{out}$, and $n_{r}$ are the numbers of hidden neurons, input neurons, membership functions, parameters in membership functions (this value is specifically intended to be two for Gaussian functions), output neurons, and rules, respectively. These values are measured for the best MLP and RBF neural networks, which are equal to 84 and 616, respectively. To calculate this value for ANFIS, we have: number of input neurons (11), number of membership functions (11), number of parameters in membership functions (2), number of output neurons (1), and number of rules (3). By placing these values in Equation (16), the value 245 is obtained for the desired parameter [53].

To determine the threshold value of the total number of adjustable parameters, the value of another parameter that is presented in the following equation must be determined:

(17) $$NPAP=\frac{1}{2}\left(N_{Training}\right)$$

The amount of $N_{Training}$ is equal to the amount of training data. This value is 296.5 for the data of this study (593 divided by 2. See Table 8 for more information). According to the literature, if NPAP is lower than TNAP, there will be no overfitting [53,54].

From the experiments performed, it can be clearly stated that the MLP neural network and the superior ANFIS neural network do not encounter any kind of overfitting in this study, but the introduced superior RBF network may have overfitting.

## 6. Relevancy Factor Evaluation

It was concluded that the introduced networks have good accuracy in predicting EOR data, for which the MLP network was introduced as the top network. In the following section, the impact of each input on the output (EOR) is measured.

(18) $$r=\frac{\sum_{i=1}^{n}\left(X_{k‚i}-\bar{X_{k}}\right)\left(Y_{i}-\bar{Y}\right)}{\sqrt{\sum_{i=1}^{n}{\left(X_{k‚i}-\bar{X_{k}}\right)}^{2}\sum_{i=1}^{n}{\left(Y_{i}-\bar{Y}\right)}^{2}}}$$

where $X_{k‚i}$ and $\bar{X_{k}}$ designate the ith value of the kth input variable and the average value of the kth input variable, respectively; $Y_{i}$ indicates the ith predicted EOR value, $\bar{Y}$ denotes the mean value of predicted values of EOR, and finally n is the amount of data in the gathered dataset. On the other hand, the value of the relevancy factor is defined in the range between −1 and +1. The closer the value of r is to +1, the more positive the effect, and the closer the value of r is to −1, the more negatively it affects the network.

Relevancy factor values for each input are presented in Table 9. Accordingly, API gravity, salinity, permeability, porosity, and salt concentration have the greatest impact on EOR. It should be noted that these cases can only be expressed for the data collected in these articles that their specifications can be seen in Table 1.

## 7. Conclusions

In this paper, MLP, RBF, and ANFIS neural networks based on subtractive clustering of EOR data using existing polymer, rock, and fluid properties, including polymer concentration, salt concentration, rock type, initial oil saturation, porosity, permeability, pore volume flooding, temperature, API gravity, molecular weight of the polymer, and salinity, were used to predict the EOR performance of HPAM polymer flooding. All the proposed models had a very high accuracy (R^2^ = 0.9990 and RMSE = 0.0002 for MLP, R^2^ = 0.9973 and RMSE=0.0008 for RBF, and R^2^ = 0.9729 and RMSE = 0.0150 for ANFIS neural network) in predicting the data, however, the MLP was the top network. Finally, by using overfitting prevention methods and testing whether the networks were overfitted or not, the networks were evaluated. It can be also clearly stated that the MLP neural network is valid in all respects to predict data inside and outside the network built-in range. Next, through relevancy factor evaluation, the parameters which have the greatest impact on EOR performance of polymer flooding were shown to be API gravity, salinity, permeability, porosity, and salt concentration. The results emphasized that by using the proposed model, the performance of HPAM polymer flooding in a special reservoir can be well evaluated before carrying out any lab-scale experiments or field-scale operations.

## Figures and Tables

**Table 1:** Features of the present study’s input variables.

Sample Number	Polymer Type	Polymer Concentration (ppm)	Salt Concentration (ppm)	Rock Type	Initial Oil Saturation	Porosity	Permeability (md)	PV (cm^3^)	Temperature (°C)	API	Molecular Weight of the Polymer (g/mol)	Salinity
1	Flopaam 3630S (SNF Floerger) polyacrylamide	300	3600	Sandstone	78	18.43–19.04	84.61–117.43	0.37–5.11	22	29.29	2 × 10^7^	High Saline
2	viscoelastic Alcoflood 935 polymer (Ciba Specialty Canada Inc., ON, Canada)	6000	10,000	Sandstone	82–88	21–22	202–219	0.04–2.92	25	31.14–36.95	9 × 10^6^	Low Saline
3	FLOPAAM 3430	2000	15,000	Sandstone	55	19.4	20	0.03–0.76	27	31.14	1 × 10^8^	Low Saline
4	HPAM	2000	0	Carbonate	89	24.2	301	0.1–1.25	27	34	6 × 10^6^	Fresh Water
5	Polyacrylamide (PAM)	2000	3276–32,754	Sandstone	72.55–75.47	25.73–28.12	212.69–240.84	0.07–2.12	70	39.3	1 × 10^4^	High Saline
6	HPAM	3100–3200	21,500	Sandstone	76.2	18.2	282	0.09–1.2	25	17	1.2 × 10^7^	Fresh Water

**Table 2:** Sensitivity analysis of MLP network with Levenberg–Marquardt backpropagation training algorithm.

Number of Hidden Neurons	Data Type	$R^{2}$	MSE	RMSE	AARD	μ	σ
2	Training	0.9850	0.0032	0.0566	194.5814	0.0257	0.0505
	Validation	0.9818	0.0039	0.0632	49.1834	0.0320	0.0547
	Testing	0.9801	0.0041	0.0640	32.2844	0.0209	0.0608
	All Data	0.9837	0.0034	0.0591	148.4453	0.0265	0.0529
5	Training	0.9977	0.0013	0.0361	53.8793	0.0307	0.0190
	Validation	0.9983	0.0017	0.0415	9.6962	0.0323	0.0260
	Testing	0.9973	0.0007	0.0278	3.5931	0.0168	0.0222
	All Data	0.9978	0.0018	0.0426	39.7145	0.0364	0.0222
**6**	**Training**	**0.9990**	**0.0002**	**0.0168**	**15.7565**	**0.0114**	**0.0123**
	**Validation**	**0.9988**	**0.0001**	**0.0135**	**5.6454**	**0.0026**	**0.0133**
	**Testing**	**0.9990**	**0.0002**	**0.0172**	**58.3171**	**0.0027**	**0.0170**
	**All Data**	**0.9990**	**0.0002**	**0.0164**	**20.6220**	**0.0100**	**0.0130**
9	Training	0.9993	0.0000	0.0085	0.0151	0.0005	0.0085
	Validation	0.9990	0.0001	0.0126	20.1423	0.0041	0.0119
	Testing	0.9992	0.0117	0.0117	5.5036	0.0067	0.0095
	All Data	0.9993	0.0001	0.0103	3.8347	0.0047	0.0091
14	Training	0.9990	0.0002	0.0151	2.7835	0.0108	0.010
	Validation	0.9990	0.0003	0.0174	1.1617	0.0138	0.0106
	Testing	0.9990	0.0008	0.0293	6.8770	0.0269	0.0117
	All Data	0.9990	0.0007	0.0278	0.7434	0.0255	0.0110
17	Training	0.9996	0.0000	0.0068	2.0459	0.0020	0.0067
	Validation	0.9996	0.0001	0.0126	7.2944	0.0099	0.0078
	Testing	0.9995	0.0002	0.0151	3.1677	0.0131	0.0075
	All Data	0.9996	0.0001	0.0127	0.8136	0.0105	0.0071

**Table 3:** Evaluation of the efficiency of different MLP neural network training methods.

Training Algorithm	MSE	RMSE	$R^{2}$	Elapsed Time (s)
Bayesian regulation backpropagation	0.001954	0.044213	0.99822	1.620583
Conjugate gradient backpropagation with Powell–Beale restarts	0.00876	0.062257	0.99494	2.083174
**Levenberg–Marquardt back propagation**	**0.002479**	**0.019794**	**0.99829**	**1.455637**
Gradient descent backpropagation	0.181400	0.425920	0.17097	0.065064
Gradient descent with adaptive learning rate backpropagation	0.008550	0.092469	0.94342	1.035535
Batch training with weight/bias learning rules	0.006039	0.077710	0.95544	3.626539
One-step secant backpropagation	0.012979	0.113930	0.99444	1.381093
Sequential order weight/bias training	0.011043	0.105090	0.91877	2.744659

**Table 4:** Sensitivity analysis for the distribution of data types.

Data Type	(%)	$R^{2}$	MSE	RMSE	AARD	μ	σ
Training	80	0.9987	0.0008	0.0284	10.0495	0.0253	0.0128
Validation	10	0.9988	0.0001	0.0133	10.5108	0.0070	0.0114
Testing	10	0.9968	0.0009	0.0309	40.8921	0.0252	0.0180
**Training**	**70**	**0.9990**	**0.0002**	**0.0168**	**15.7565**	**0.0114**	**0.0123**
**Validation**	**15**	**0.9988**	**0.0001**	**0.0135**	**5.6454**	**0.0026**	**0.0133**
**Testing**	**15**	**0.9990**	**0.0002**	**0.0172**	**58.3171**	**0.0027**	**0.0170**
Training	60	0.9991	0.0002	0.0167	1.1088	0.0100	0.0134
Validation	20	0.9984	0.0004	0.0207	6.2579	0.0114	0.0173
Testing	20	0.9982	0.0019	0.0436	382.0992	0.0360	0.0247
Training	50	0.9979	0.0013	0.0360	18.7630	0.0300	0.0199
Validation	25	0.9959	0.0006	0.0257	6.6518	0.0144	0.0212
Testing	25	0.9950	0.0049	0.0684	139.5505	0.0540	0.0421
Training	40	0.9982	0.0007	0.0278	60.9115	0.0206	0.0186
Validation	30	0.9966	0.0014	0.0387	3.4631	0.0313	0.0227
Testing	30	0.9943	0.0021	0.0465	45.5541	0.0365	0.0288
Training	70	0.9983	0.0016	0.0411	51.0327	0.0347	0.0221
Validation	20	0.9977	0.0021	0.0461	1.7933	0.0390	0.0247
Testing	10	0.9982	0.0007	0.0266	206.1784	0.0157	0.0215
Training	70	0.9985	0.0012	0.0348	45.5224	0.0290	0.0193
Validation	10	0.9984	0.0007	0.0270	195.1773	0.0226	0.0155
Testing	20	0.9982	0.0022	0.0477	7.2765	0.0419	0.0229

**Table 5:** Sensitivity analysis of RBF network.

Number of Hidden Neurons	Spread	Data Type	$R^{2}$	MSE	RMSE	AARD	μ	σ
22	1.1	Training	0.9913	0.0017	0.0412	123.8424	0.0271	0.0311
		Testing	0.9904	0.0019	0.0444	229.5288	0.0301	0.0327
		All Data	0.9910	0.0018	0.0425	155.5358	0.0286	0.0315
22	2	Training	0.9878	0.0015	0.0393	161.0398	0.0068	0.0387
		Testing	0.9858	0.0020	0.0456	329.8626	0.0221	0.0399
		All Data	0.9872	0.0016	0.0403	211.6667	0.0067	0.0398
22	10	Training	0.9818	0.0025	0.0208	156.0130	0.0212	0.0461
		Testing	0.9765	0.0024	0.0495	243.5169	0.0115	0.0482
		All Data	0.9804	0.0025	0.0509	182.2538	0.0202	0.0467
30	1.1	Training	0.9941	0.0006	0.0256	89.0743	0.0032	0.0254
		Testing	0.9950	0.0006	0.0257	158.5864	0.0063	0.0249
		All Data	0.9944	0.0007	0.0265	109.9198	0.0081	0.0252
**44**	**1.1**	**Training**	**0.9983**	**0.0003**	**0.0196**	**5.5895**	**0.0127**	**0.0149**
		**Testing**	**0.9973**	**0.0008**	**0.0283**	**6.0256**	**0.0191**	**0.0209**
		**All Data**	**0.9980**	**0.0007**	**0.0282**	**2.1064**	**0.0212**	**0.0185**
45	1.1	Training	0.9986	0.0002	0.0164	6.7843	0.0089	0.0138
		Testing	0.9979	0.0003	0.0195	13.4048	0.0118	0.0155
		All Data	0.9970	0.0004	0.0212	7.3379	0.0111	0.0181
50	1.1	Training	0.9987	0.0008	0.0287	3.7032	0.0222	0.0182
		Testing	0.99801	0.0029	0.0539	285.5218	0.0418	0.0340
		All Data	0.9985	0.0029	0.0540	83.0302	0.0444	0.0307

**Table 6:** The performance of different types of ANFIS.

ANFIS Type	Max Epoch	Data Type	$R^{2}$	MSE	RMSE	AARD
Grid Partitioning	5	Training	0.9557	0.0073	0.0855	105.3691
		Testing	0.9562	0.0088	0.0941	267.7566
		All Data	0.9559	0.0077	0.0880	154.0662
**Subtractive Clustering**	**100**	**Training**	**0.9749**	**0.0146**	**0.1210**	**195.5981**
		**Testing**	**0.9678**	**0.0135**	**0.1162**	**5.3421**
		**All Data**	**0.9729**	**0.0150**	**0.1226**	**138.5438**
FCM Clustering	100	Training	0.9519	0.0059	0.0771	157.8325
		Testing	0.9494	0.0057	0.0761	293.8182
		All Data	0.9511	0.0060	0.0779	198.6106

**Table 7:** Sensitivity analysis of ANFIS network based on subtractive clustering.

Radius	Initial Step Size	Step Size Decrease Rate	Step Size Increase Rate	Data Type	$R^{2}$	MSE	RMSE	AARD	μ	σ
0.333	4	11	13	Training	0.9376	0.0119	0.1039	37.3869	0.0138	0.1085
				Testing	0.9408	0.0091	0.0957	472.6515	0.0395	0.0873
				All Data	0.9385	0.0113	0.1063	167.9149	0.0129	0.1055
0.333	14	11	13	Training	0.9261	0.0156	0.1249	275.7756	0.0447	0.1167
				Testing	0.9249	0.0194	0.1395	62.7544	0.0626	0.1249
				All Data	0.9256	0.0185	0.1361	211.8944	0.0592	0.1226
0.333	4	23	13	Training	0.9410	0.0102	0.1010	43.9129	0.0057	0.1010
				Testing	0.9403	0.0112	0.1062	500.3728	0.0262	0.1031
				All Data	0.9409	0.0109	0.1046	180.7970	0.0193	0.1028
**0.333**	**4**	**23**	**20**	**Training**	**0.9749**	**0.0146**	**0.1210**	**195.5981**	**0.1030**	**0.0635**
				**Testing**	**0.9678**	**0.0135**	**0.1162**	**5.3421**	**0.0969**	**0.0643**
				**All Data**	**0.9729**	**0.0150**	**0.1226**	**138.5438**	**0.1044**	**0.0643**
4	4	23	20	Training	0.9448	0.0173	0.1316	202.9277	0.0750	0.1028
				Testing	0.9426	0.0146	0.1211	9.4858	0.0588	0.1061
				All Data	0.9441	0.0170	0.1307	144.9179	0.0729	0.1086
0.4	4	23	20	Training	0.9753	0.0055	0.0746	228.8593	0.0473	0.0605
				Testing	0.9659	0.0074	0.0865	22.1922	0.0552	0.0667
				All Data	0.9727	0.0069	0.0835	166.8836	0.0536	0.0641
0.3	4	23	20	Training	0.9330	0.0199	0.1411	134.4072	0.0951	0.1043
				Testing	0.9241	0.0262	0.1621	185.1166	0.0121	0.1076
				All Data	0.9299	0.0264	0.1626	149.6141	0.1196	0.1101

**Table 8:** Properties of the optimized MLP model.

Parameter	Value or Description
Amount of all/training/validating/testing data	847/593/127/127
Number of input/output variables	11/1
Training method	Levenberg–Marquardt backpropagation
Number of neurons in the hidden layer	6
Number of hidden layers	1
Number of neurons in the input/output layer	11/1
Transfer function in the hidden/output layer	Tangent sigmoid/Linear
Number of epochs	1000

**Table 9:** Relevancy factor to predict EOR.

Input Variable	MLP	RBF	ANFIS	Original EOR
Polymer Concentration	−0.1035	−0.1122	−0.1087	−0.1058
Salt Concentration	0.7592	0.7593	0.7590	0.7598
Rock Type	−0.2933	−0.2835	−0.2809	−2928
Initial Oil Saturation	−0.2930	−0.2945	−0.2949	−0.2942
Porosity	−0.7848	−0.7839	−0.7826	−0.7851
Permeability	0.8512	0.8529	0.8594	0.8519
Pore Volume flooding	−0.2335	−0.2370	−0.2417	−0.2343
Temperature	−0.5758	−0.5731	−0.5730	−0.5749
API of the Petroleum	−0.9064	−0.9067	−0.9048	−0.9070
Molecular Weight of the Polymer	−0.2143	−0.2172	−0.2160	−0.2155
Salinity	−0.8682	−0.8698	−0.8727	−0.8688
